# Supplementary material for: Beeswax Alcohol Prevents Low-Density Lipoprotein Oxidation and Demonstrates Antioxidant Activities in Zebrafish Embryos and Human Subjects: A Clinical Study
Source: Curr Issues Mol Biol. 2024 Jan 2;46(1):409–29. doi: 10.3390/cimb46010026 (PMC10813917; doi:10.3390/cimb46010026)
Supplement: Supplementary file 1 [file cimb-46-00026-s001.zip › cimb-2730485-supplementary.pdf]

Supplementray material

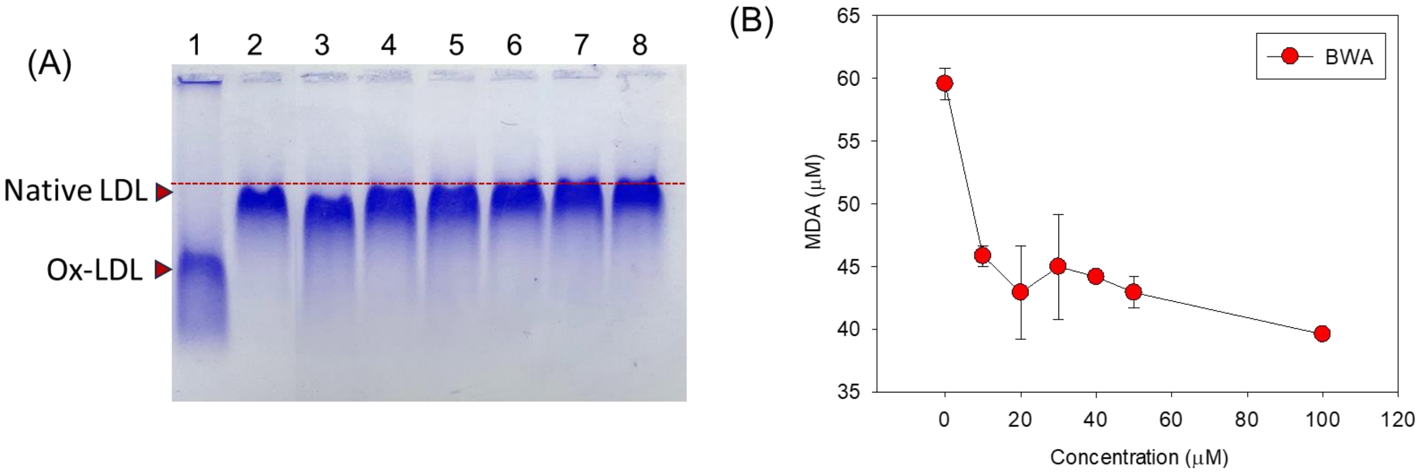

Supplementary Figure S1. Effect of beeswax alcohol (BWA) on $CuSO_4$ induced oxidation of LDL. **(A)** Electrophoretic mobility of apo-B fraction of LDL. Lanes 1 represent LDL+$CuSO_4$ (Ox LDL), Lane 2 represent Native LDL, Lane 3, 4, 4, 5, 7, 8 represent LDL+$CuSO_4$ treated with 10, 20, 30, 40, 50 and 100 μM BWA. Electrophoresis was performed on 0.5% agarose gel using Tri-EDTA buffer (pH 8.0) at a constant voltage (50 V). The red dotted line indicates a similar electromobility front. **(B)** Quantification of $CuSO_4$-induced LDL oxidation in the presence and absence of BWA. The LDL oxidation was quantified by thiobarbituric acid reactive substance (TBARS) assay using malondialdehyde (MDA) as reference.

Supplementary Figure S2.

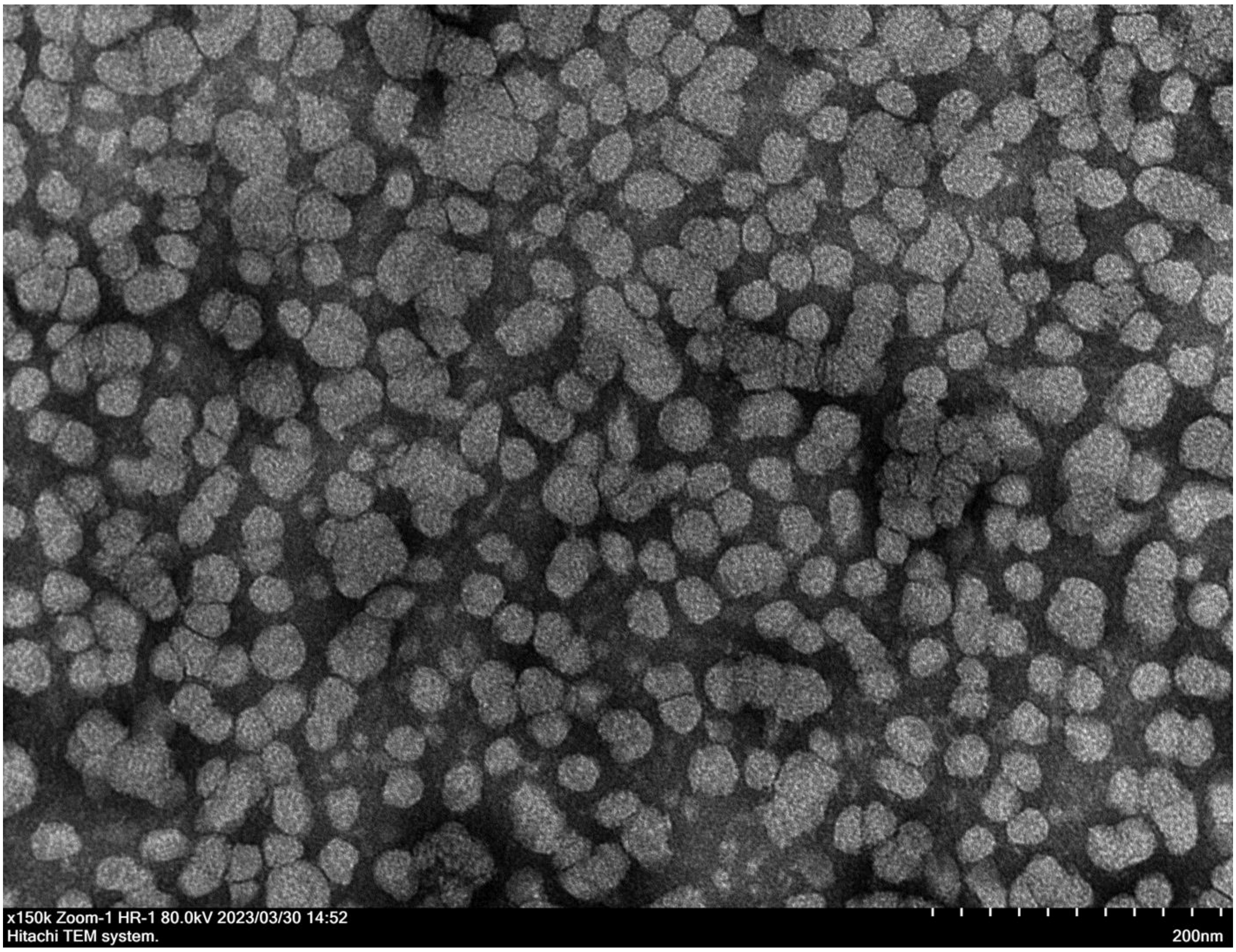

Supplementary Figure S3.

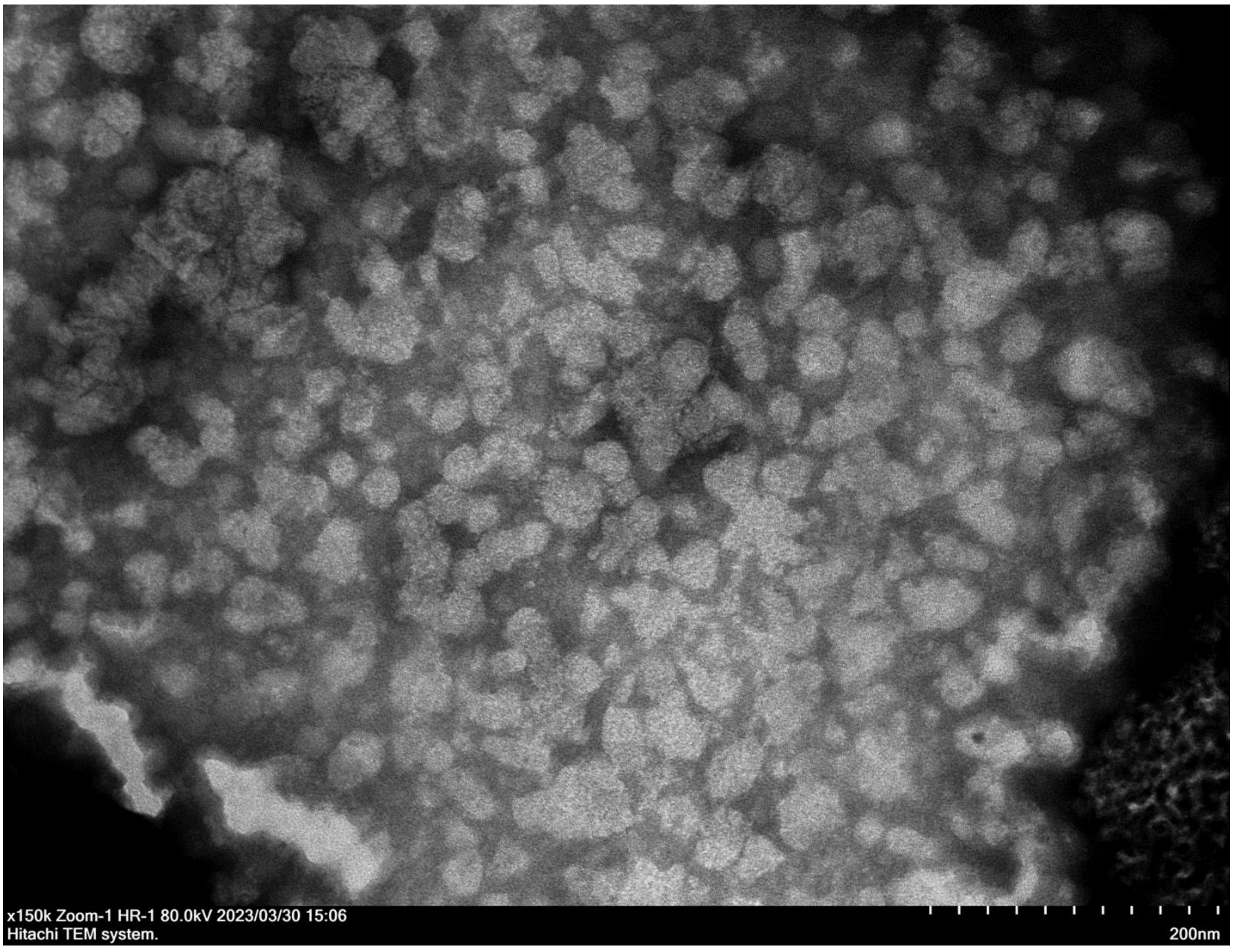

Supplementary Figure S4.

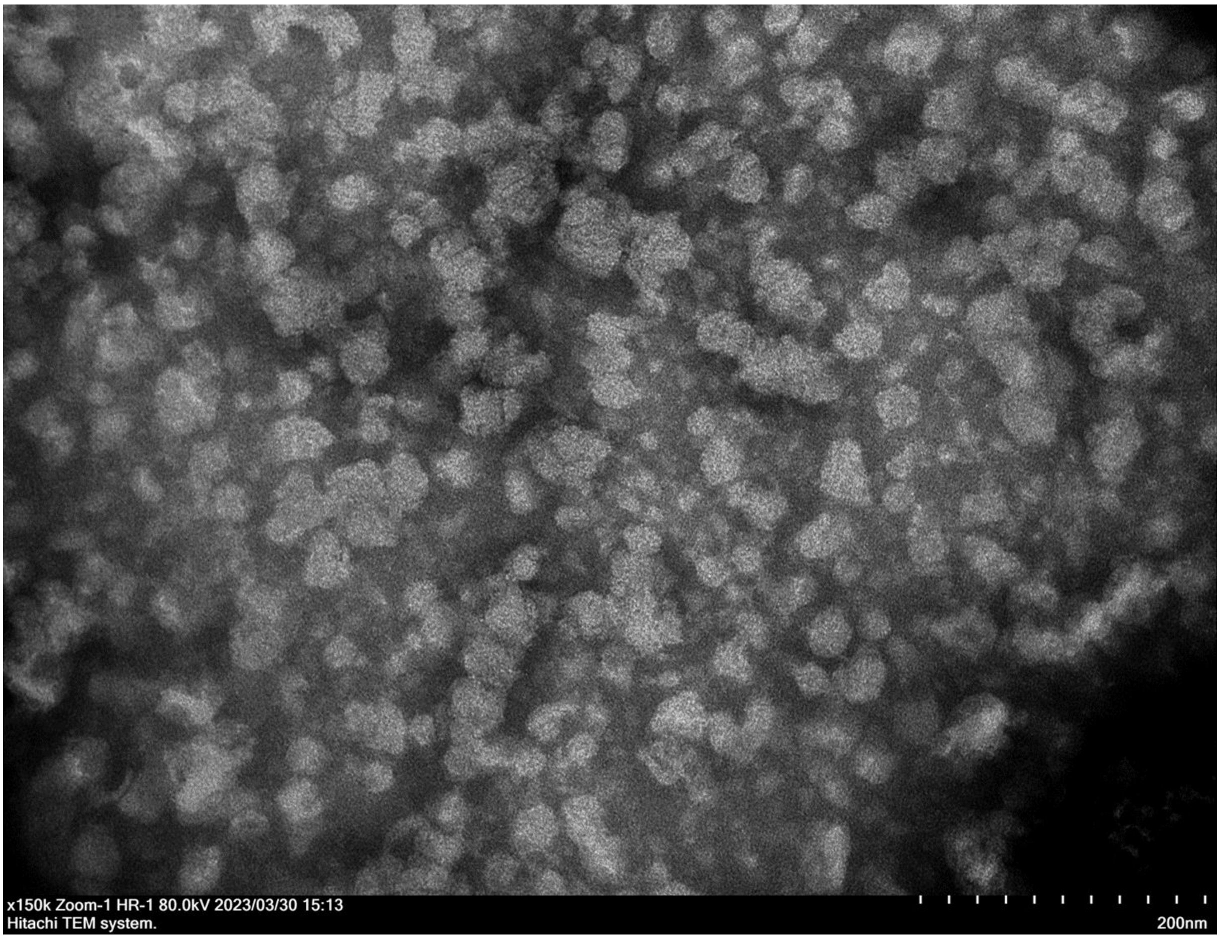

Supplementary Figure S5.

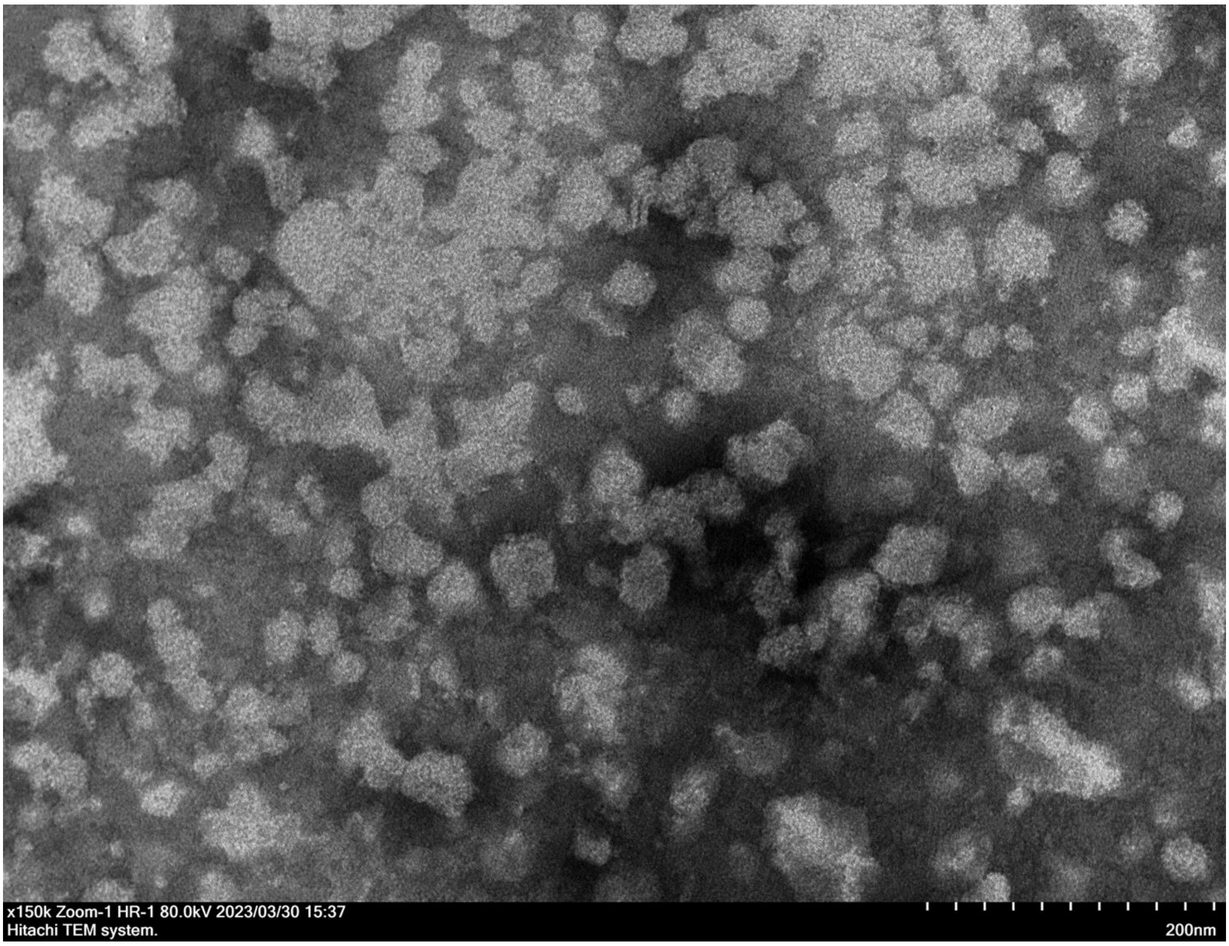

Supplementary Figure S6.

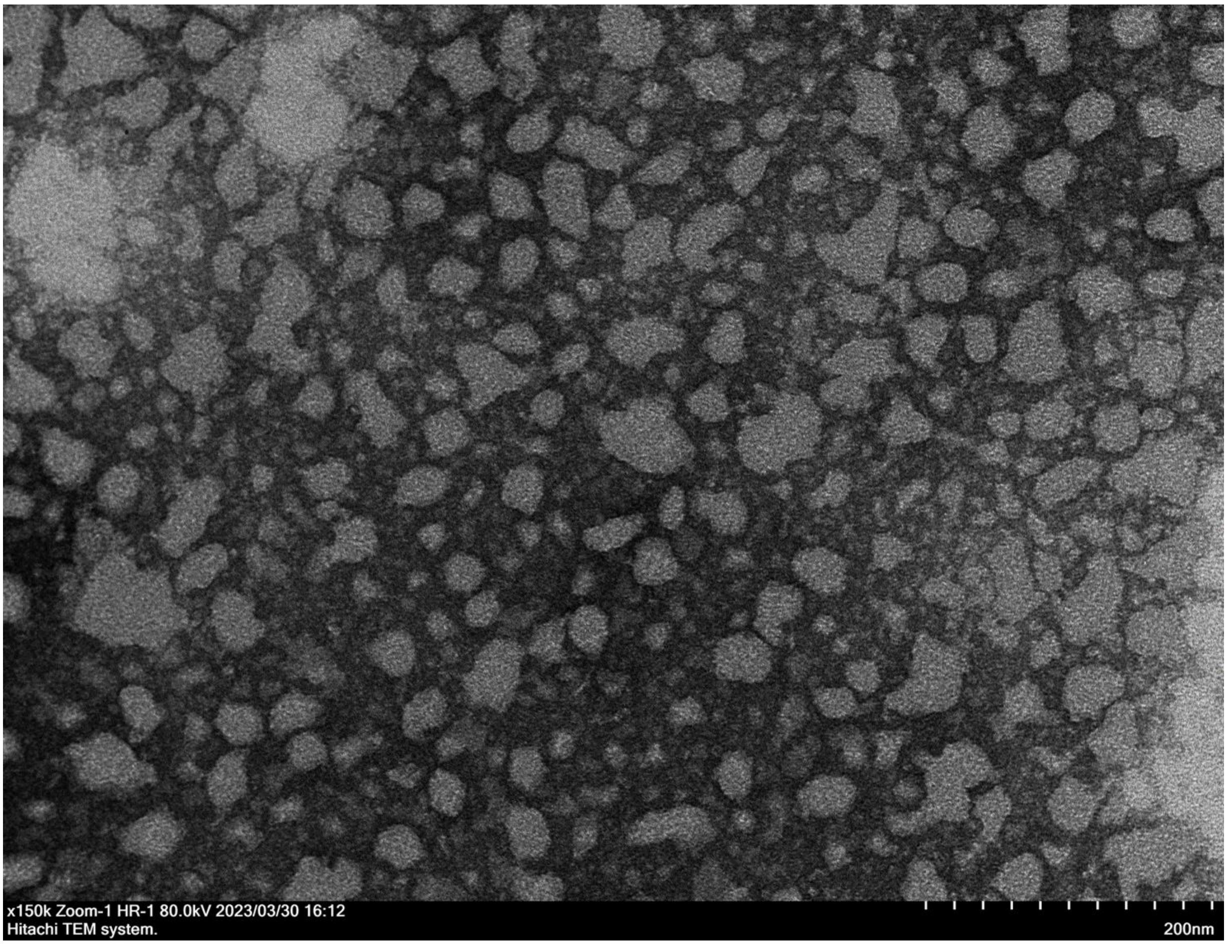

Supplementary Figure S7.

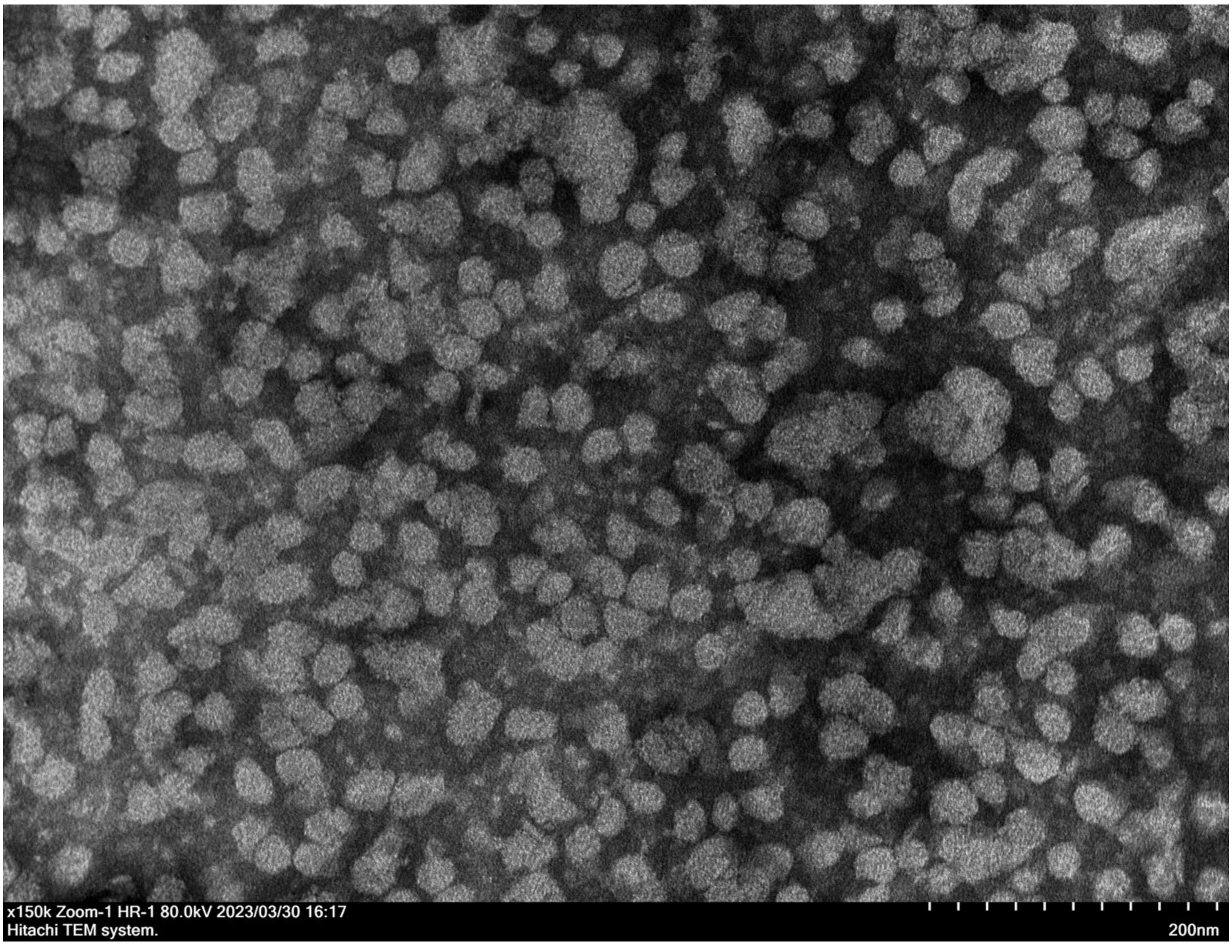

Supplementary Figures S2–S7. Magnified image representing the effect of beeswax alcohol (BWA) on size and structural alteration of LDL probed by transmission electron microscope (TEM). **Figure S2.** TEM images of native LDL. **Figure S3.** TEM images of LDL treated with $CuSO_4$. **Figure S4,S5,S6,S7** LDL treated with $CuSO_4$ in the presence of 10, 20, 30, 40 BWA μM BWA, respectively.
